# Mechanisms of prey division in striped marlin, a marine group hunting predator

**DOI:** 10.1038/s42003-022-03951-3

**Published:** 2022-10-31

**Authors:** M. J. Hansen, S. Krause, F. Dhellemmes, K. Pacher, R. H. J. M. Kurvers, P. Domenici, J. Krause

**Affiliations:** 1 Department of Fish Biology, Fisheries and Aquaculture, Leibniz Institute of Freshwater Ecology and Inland Fisheries, Müggelseedamm 310, 12587 Berlin, Germany; 2 Department of Electrical Engineering and Computer Science, Lübeck University of Applied Sciences, 23562 Lübeck, Germany; 3 Faculty of Life Science, Humboldt-Universität zu Berlin, Invalidenstrasse 42, 10115 Berlin, Germany; 4 Center for Adaptive Rationality, Max Planck Institute for Human Development, Lentzeallee 94, 14195 Berlin, Germany; 5 IBF-CNR, Consiglio Nazionale delle Ricerche, Area di Ricerca San Cataldo, Via G. Moruzzi N°1, 56124 Pisa, Italy; 6 IAS-CNR, Località Sa Mardini, 09170 Torregrande, Oristano Italy; 7 Cluster of Excellence “Science of Intelligence,” Technical University of Berlin, Marchstr. 23, 10587 Berlin, Germany; 8 Present Address: Faculty of Life Science, Humboldt-Universität zu Berlin, Invalidenstrasse 42, 10115 Berlin, Germany

**Keywords:** Behavioural ecology, Social evolution

## Abstract

Many terrestrial group-hunters cooperate to kill prey but then compete for their share with dominance being a strong predictor of prey division. In contrast, little is known about prey division in group-hunting marine predators that predominately attack small, evasive prey (e.g. fish schools). We identified individual striped marlin (*Kajikia audax*) hunting in groups. Groups surrounded prey but individuals took turns attacking. We found that competition for prey access led to an unequal division of prey among the predators, with 50% of the most frequently attacking marlin capturing 70–80% of the fish. Neither aggression, body size nor variation in hunting efficiency explained this skewed prey division. We did find that newly arrived groups of marlin gained on average more access to the prey. This raises the possibility that newly arrived marlin were hungrier and more motivated to feed. However, this result does not necessarily explain the unequal prey division among the predators because the skew in prey captures was found at the level of these groups. Dynamic prey division is probably widespread but under-reported in marine group-hunters and the inability of individuals to monopolize prey until satiation likely reduces the importance of social hierarchies for prey division.

## Abstract (web summary)

Striped marlin use a dynamic prey division method when hunting as a group, taking turns to feed but without doing so equally.

## Introduction

Resource competition influences the distribution and behaviour of species, and how group-living animals divide resources is a driving force behind the evolution of sociality^1,2^. For group-hunting predators, the division of prey between group members affects the degree to which individuals benefit from hunting in a group. However, quantifying inter-individual variation in prey access and intake remains a challenge across many group-hunting species^3–6^, primarily because tracking the behaviour of multiple individuals over long periods of the hunt, up to and including prey consumption, is rarely achieved^7,8^. As a consequence, our knowledge of the potential factors (e.g. dominance, motivation, hunting skill or social roles) determining inter-individual variation in prey access and intake is still in its infancy. This is especially relevant because the wide diversity of prey and predator types suggests there will be significant differences between group-hunting systems.

Group-hunting research has traditionally focused on terrestrial study systems. Group-hunting terrestrial predators commonly target relatively large prey (i.e., typically prey-predator length ratios > 1) and divide their prey (in the form of a carcass) after the hunt has been completed^3,4^. Prey division in terrestrial systems is predominantly governed by dominance hierarchy, kinship and social bonds which determine the feeding order to a shared kill^2,9–14^. For example, in lions, *Panthera leo*, males supplant females from carcasses and gain priority access to the resource (with females gaining equal access among themselves)^9,15,16^. Alpha coyotes (*Canis latrans*) also enjoy priority access to carcasses, allowing them to feed on nutritious organs and large muscle tissues, thereby achieving higher biomass intake than lower-ranked individuals^4^. Predators that feed secondarily not only consume less nutritious tissues, but also run a higher risk of having their meal kleptoparasitised before they have fed.

Relatively little is known about prey division in group-hunting predators in marine environments. Many marine predator species hunt in groups (such as some cetaceans, some teleost fishes and some sharks), attacking schooling prey substantially smaller than themselves (prey-predator length ratios < 0.1)^17–21^. This creates very different conditions for prey division compared to terrestrial systems because, firstly, although the total resource itself can be large, it is composed of multiple prey items that can be divided between the predators during the hunt. Secondly, in combination with its large size, the collective evasive behaviour of the prey makes it hard for a single predator to monopolise the total resource or otherwise exert control over predator access^22^. Consequently, it has been suggested that marine group-hunting predators should show less aggression, both within- and between predator species, and lower variation in resource consumption between group members compared to their terrestrial counterparts (where high-ranked individuals can control access to a carcass)^2^. However, given that each marine group-hunting predator of schooling fish must still catch its own prey items, we might also expect individual hunting skills and attack effort to have a direct effect on the success of each single predator. The hunting success of terrestrial predators, on the other hand - as well as orca (*Orcinus orca*) that hunt large whales^23^ - often depends on a collective effort, where a single large prey must be subdued together^24^.

The individual intakes of group hunters have traditionally been estimated by dividing the difference in known—or estimated—mass of the carcass, before and after feeding, by the number of predators present or their time spent feeding from the carcass. This often requires controlling for the state of the carcass (e.g., ‘intact with viscera’) because body parts can have different nutritional and energetic composition and be consumed at different rates^3,4,15,25–30^. This constitutes a challenge to estimate individuals’ relative and absolute energetic gains from a hunt^7,8^. Nevertheless, studies have estimated per capita food intake (using the above methods) and have also used the calculations to expose situations where social predators form groups for reasons other than maximising individual food intake^25,31^. Schmidt & Mech (1997)^31^, for example, showed that food intake decreases with group size in wolf (*Canis lupus*) packs and proposed groups are formed due to kin-selection^31^. Chakrabarti & Jhala (2017)^28^ found very unequal resource acquisition within male Asiatic lion (*Panthera leo persica*) coalitions (dominant lions get 47% more food than their subordinates) and concluded that coalitions were formed to increase mating opportunities and not to increase individual food intake^28^.

The relationship between group-hunting and individual intakes are even less well explored in marine predators which hunt schools of fish. Without controlling for individual identity, Major et al. (1978)^32^ found that the proportion of prey captured by giant trevally (*Caranx ignobilis*) increased when predators hunted with conspecifics^32^. However, this study had to be conducted in semi-wild conditions which restricted the movements of both predator and prey. Thiebault et al. (2016)^33^ used underwater video of wild group-hunts and found that for Cape gannet (*Morus capensis*) feeding success increased when attacks were close in time to other predators, but again, without controlling for individual identity of the predators^33^. These two studies are a rarity, however, as direct observation of captures is usually not possible in marine systems. Therefore, other proxies have been used to infer capture success. Benoit-Bird & Au (2009)^18^, for example, used prey density and Handegard et al. (2012)^34^ used distance to prey as proxies for feeding success^18,34^. Benoit-Bird & Au (2009)^18^ showed that spinner dolphins (*Stenella longirostris*) collectively herd and condense their prey before encircling and taking turns attacking. Handegard et al. (2012)^34^ showed that spotted sea-trout (*Cynoscion nebulosus*) formed lines when attacking prey schools, effectively fragmenting the school, which allowed predators to get closer to the prey. These latter two studies used high-resolution sonar to track the movements of multiple predators and prey, showing benefits of group-hunting. However, none of these studies assessed how the resource was divided between the group-hunting predators. To achieve this, two major challenges need to be overcome. Firstly, individual identities of the hunting party must be tracked (this is especially difficult under water as aquatic predators often constitute open groups with fluid memberships and travel great distances). Secondly, determining the capture success of single individuals often requires close-up (and often high-speed) video footage and, in the absence of remote sensing equipment attached to the entire hunting party, a close presence of the observers without disturbing the hunt. In a rare exception, Gazda et al. (2016)^19^ observed that bottlenose dolphins (*Tursiops truncatus*) which take up the social role of “drivers” and push prey fish towards conspecifics acting as a “barrier”, had the highest foraging success^19^. Overall, however, there is a lack of studies tracking the temporal dynamics of marine group-hunting in the wild from an individual-level perspective^35^. Hence there is little information on how resources are divided in such systems.

In this study, we investigated the group-hunting behaviour of striped marlin (*Kajikia audax*) attacking schools of fish in the open ocean. Groups of marlin surround the fish school and attack schooling prey one at a time by making repeated high-speed attacks (‘dash sequence’, Supplementary Video 1), occasionally using their bill to capture prey^36^. Across two years, we identified 54 individual striped marlin (34 individuals in 2018 and 20 individuals in 2019) and investigated how these predators divided two different schools of prey fish (*Sardinops sagax caerulea* in 2018 and *Scomber japonicus* in 2019) among themselves - by recording individuals’ attack effort and capture success. We found that prey division between marlin was unequal but surprisingly not linked to body size differences or aggression. We found more evidence, albeit it indirect, for motivationally-driven differences in prey access compared to other potential mechanisms, including predator exhaustion.

## Results

**Fig. 1:**
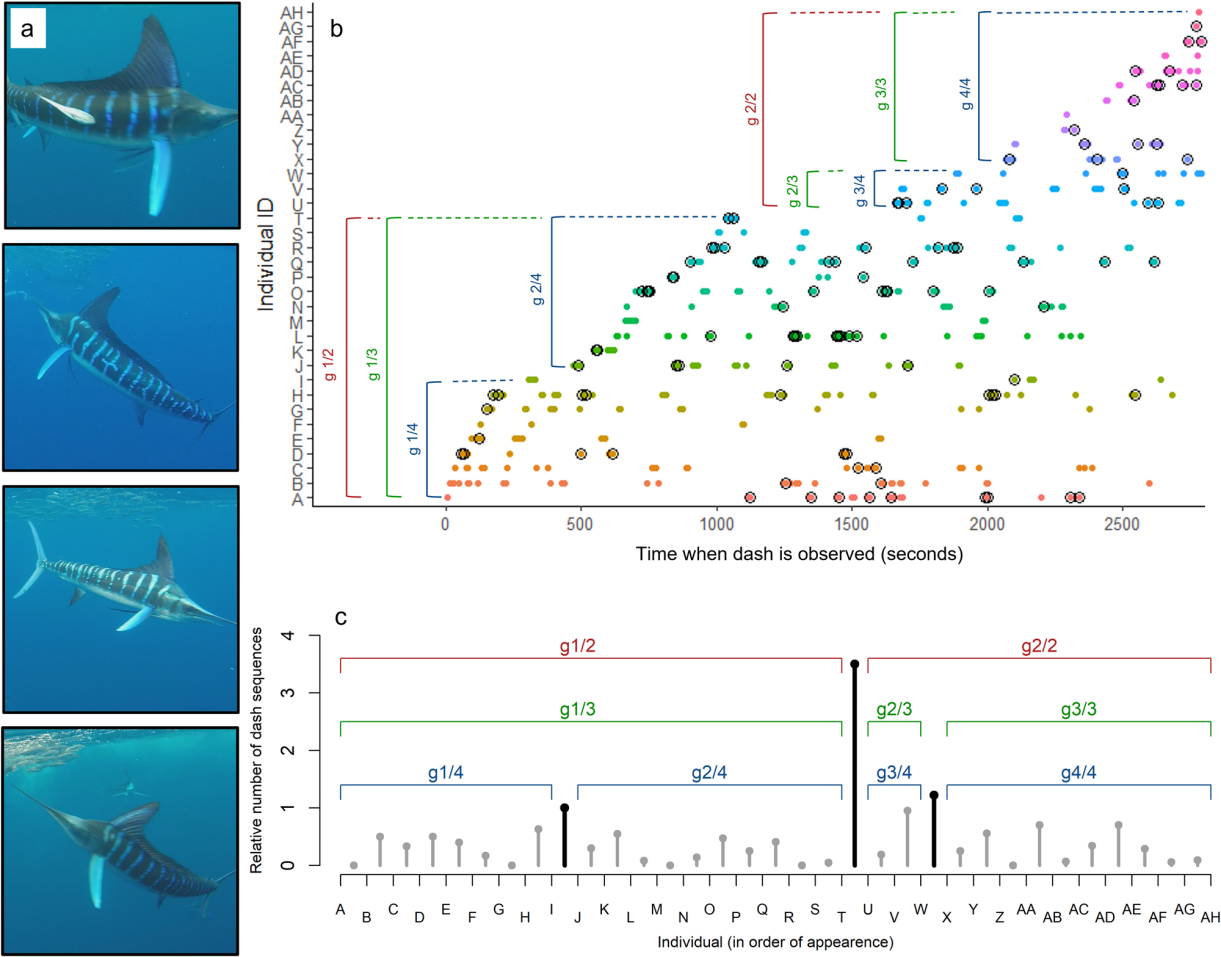
Dashes and captures of individually identified striped marlin with potential sub-grouping. **a** Examples of unique markings used for individually identifying marlin (images created by Matthew Hansen). **b** Each dot shows the timing of a dash by an individual marlin in 2018. Marlin are colour coded to differentiate between individuals. Dashes that resulted in a prey capture are encircled (○). Groups arriving at different time points are bracketed in different colours, with three potential scenarios: marlin arriving in two groups (), three groups (), or four groups (). **c** The three potential sets of arriving groups of marlin in 2018 that can be derived from the numbers of dash sequences between the appearances of individual marlin, two groups (), three groups () or four groups (). Fig. S1 shows corresponding 2019 data.

We filmed group-hunting striped marlin offshore Baja California, Mexico in November 2018 and again in 2019 as they attacked a school of prey fish (*Sardinops sagax caerulea*, *N* = approx. 140 in 2018, *Scomber japonicus* *N* = approx. 175 in 2019). We recorded marlin attacking the prey school continuously for 46 min in 2018 and 17 min in 2019. We identified individual marlin by characteristic markings such as lateral stripes and dorsal fin shape (Fig. 1a). For each individual, we scored when it attacked and whether or not the attack resulted in a prey capture (Fig. 1b). Marlin attacked schooling prey by ‘dashing’ through the school, often followed by a quick turn and reapproach of the school^36^. An individual can thus make consecutive dashes at the prey school before a different marlin initiates an attack. We defined an attack as a ‘dash sequence’: one or multiple consecutive ‘dashes’ by the same individual (2018: Mean ± SE dash sequence length: 2.4 ± 0.1 dashes, range 1–14; 2019: 1.9 ± 0.1 dashes, range 1–6; Table S1; Supplementary Video 1). We scored a total of 711 dashes (297 dash sequences) and 110 captures in 2018 (Fig. 1b; Table S1), and 350 dashes (186 dash sequences) and 58 captures in 2019.

### Subgroups and individual dash frequency

**Fig. 2:**
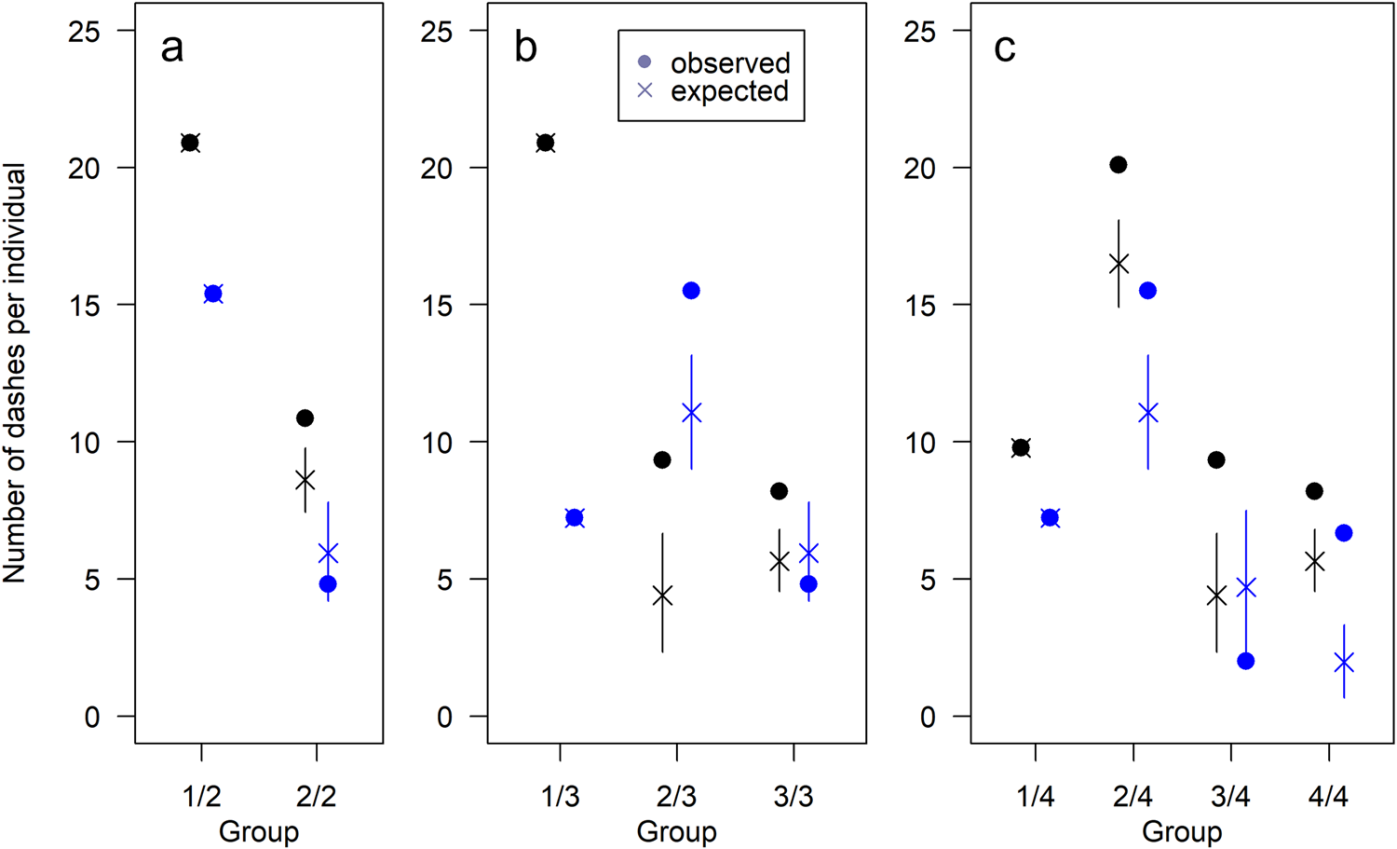
Numbers of dashes per individual marlin across sub-groups. **a**–**c** Observed (•) and expected (x) numbers of dashes per individual plus 2.5th and 97.5th percentile. for newly arrived groups in the period from their arrival until the arrival of the next subgroup, assuming the marlin arrived at the prey resource sequentially in **a** two groups, **b** three groups or **c** four groups. Individuals in newly arrived subgroups performed significantly more dashes than expected (expectation based on equal access to the prey resource for all individuals present) and thus attained greater access to the prey school than expected. Note that the perfect match between observed and expected numbers for groups 1/2, 1/3, and 1/4 is a statistical necessity as the newly arrived individuals are the only ones present. Black colours depict data for 2018 and blue for 2019.

In contrast to most terrestrial group-hunting predators where group membership is typically closed during a hunt, in marlin (and many other marine group-hunting species), group membership is more fluid, and individuals may arrive at different time points during the hunt. Therefore, we analysed when individuals first made identifiable attacks in our recording (see Methods), to be able to estimate the reward per unit effort and accurately capture the temporal dynamics of prey division. This analysis suggested that marlin arrived in different subgroups with 2, 3 or 4 subgroups being most likely both in 2018 (Fig. 1b, c), and 2019 (Fig. S1). The estimated total number of subgroups depends on different threshold values of the relative frequency of dash sequences that occur between the appearances of individual marlin (see Methods, Fig. 1b, c, Fig. S1). Comparing dash frequency of newly arriving individuals to already present individuals, we found that marlin in newly arrived subgroups performed more dashes than expected in the period from their arrival till the arrival of the next subgroup, compared to those which were already present at the sardine school (Fig. 2a–c). As arrival time differences affect individuals’ opportunities for prey access, we accounted for arrival time differences in our subsequent analyses of attack frequencies, capture success, and attack order.

### Relationship between frequency of dash sequences and captures

**Fig. 3:**
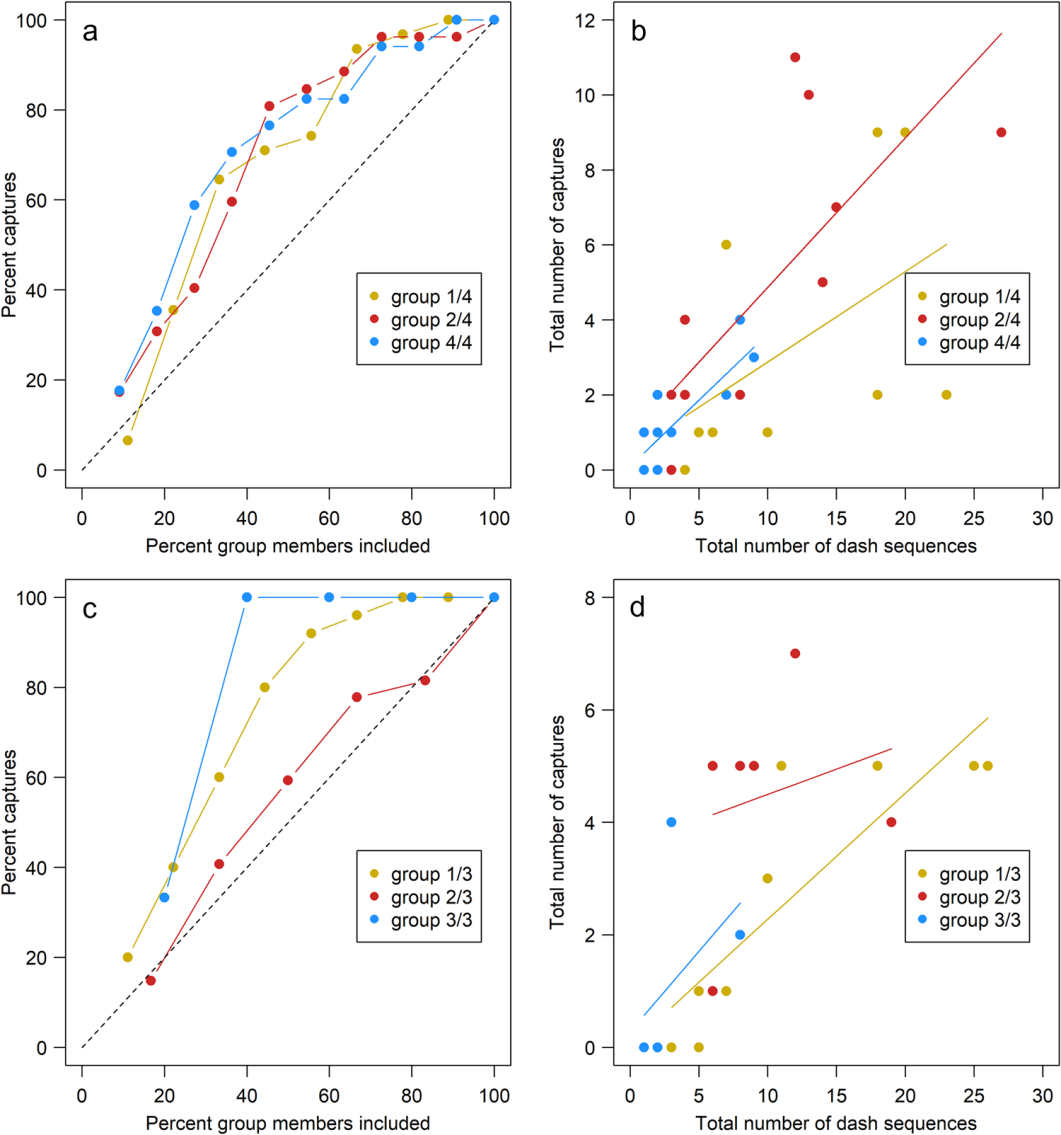
Division of prey within sub-groups. **a**, **b** 2018 data; **c**, **d** 2019 data. **a**, **c** For the scenario of four subgroups in 2018 and three subgroups in 2019 (due to very small group sizes in 2019 for the scenario of four subgroups), the relationship between the percentage of prey captured and the percentage of group members—sorted in descending order of their number of dash sequences—showing the unequal division of prey between group members. For the different subgroups— 2018: 1/4 (), 2/4 () and 4/4 (); 2019: 1/3 (), 2/3 () and 3/3 (), —approx. 70–80% of sardines are captured by less than 50% of the members. Group 3 is not shown in **a**, **b** – 2018 - because it only contained three members. Deviations from the diagonal dotted line show that the division of prey is unequal. **b**, **d** Within each of the subgroups, individuals with a higher number of dash sequences also had a higher total number of captures.

Within each of the subgroups, we ranked individuals according to their number of dash sequences and determined how this was linked to their number of captures. We found that the 50% of most frequently attacking marlin captured approximately 70–80% of the total number of captured sardines and this was independent of subgroup number (Fig. 3a, c). To investigate this result further, we tested whether individuals in subgroups differed in their number of dash sequences, and how this related to captures. The number of dash sequences differed between individuals (2018: Chi-squared test, all *p* ≤ 0.002 for all group sizes ≥ 9; 2019: *p* < 0.001 for all group sizes ≥ 9; see Table 1 for all group comparisons) and individuals’ number of dash sequences correlated positively with their number of captures (2018: Spearman: *p* < 0.026 for group sizes ≥ 9; 2019: Spearman: *p* < 0.005 for group sizes ≥ 9; Table 1; Fig. 3b, d). Thus, newly arrived subgroups performed more dashes in the period from their arrival until the arrival of the next subgroup than marlin that were already present at the sardine school. Additionally, within subgroups there was unequal division of the prey over the whole recorded hunt, with the most frequently attacking marlin capturing a proportionally greater share of the prey.

**Table 1:** Statistical results for dash and capture analysis.

	2018					2019				
Group	*N*	χ^2^ number of dash sequences		Spearman: dash sequences and captures		*N*	χ^2^ number of dash sequences		Spearman: dash sequences and captures	
		χ^2^	*p*-value	ρ	*p*-value		χ^2^	*p*-value	ρ	*p*-value
1/2	20	95.4	**<** **0.001**	0.64	**0.002**	15	66.1	**<** **0.001**	0.69	**0.004**
2/2	14	49.5	**<** **0.001**	0.76	**0.002**	5	9.6	**0.047**	0.80	0.102
1/3	20	95.4	**<** **0.001**	0.64	**0.002**	9	49.9	**<** **0.001**	0.94	**<** **0.001**
2/3	3	2.8	0.247	0.50	1	6	12.2	**0.032**	0.25	0.638
3/3	11	28.1	**0.002**	0.84	**0.001**	5	9.6	**0.047**	0.80	0.102
1/4	9	35.2	**<** **0.001**	0.73	**0.026**	9	49.9	**<** **0.001**	0.94	**<** **0.001**
2/4	11	58.6	**<** **0.001**	0.80	**0.003**	6	12.2	**0.032**	0.25	0.638
3/4	3	2.8	0.247	0.50	1	2	0.0	1	NA	NA
4/4	11	28.1	**0.002**	0.84	**0.001**	3	6.5	**0.039**	0.50	1

*P*-values (statistically significant in **bold**) for within group Chi-squared tests for uniform distribution of individual numbers of dash sequences, and Spearman correlation of the number of dash sequences and number of captures. Results are shown for the 2, 3 or 4 groups estimated to occur in 2018 and 2019. N refers to the number of individual marlin in each subgroup. For subgroups with sufficiently large sample size (n ≥ 9), there is good evidence that the number of dash sequences is not uniformly distributed and that the number of dash sequences correlates with the number of captures.

### Length of dash sequences and interruptions

Next, we assessed the mean length of dash sequences across all identified marlin attacking the same prey school, regardless of subgroup. Dash sequence length refers to the number of consecutive dashes performed by a marlin before being interrupted, it is not calculated by assessing the time spent attacking. We found evidence for inter-individual differences in the mean length of dash sequences (2018: randomisation test: *p* = 0.03; 2019: *p* = 0.02). However, these differences did not correlate with the number of captures (2018: Pearson correlation: *r* = 0.30, *p* = 0.19; 2019: *r* = −0.17, *p* = 0.55; only using individuals with 5 or more dash sequences to get robust values of the mean dash sequence length). Dash sequence length followed a geometric distribution (Fig. S3) which indicates that dash sequence length is largely a probabilistic outcome. We suggest that dash sequences were most likely terminated by external events such as the evasive behaviour of the prey school or a conspecific interrupting a dash sequence.

**Fig. 4:**
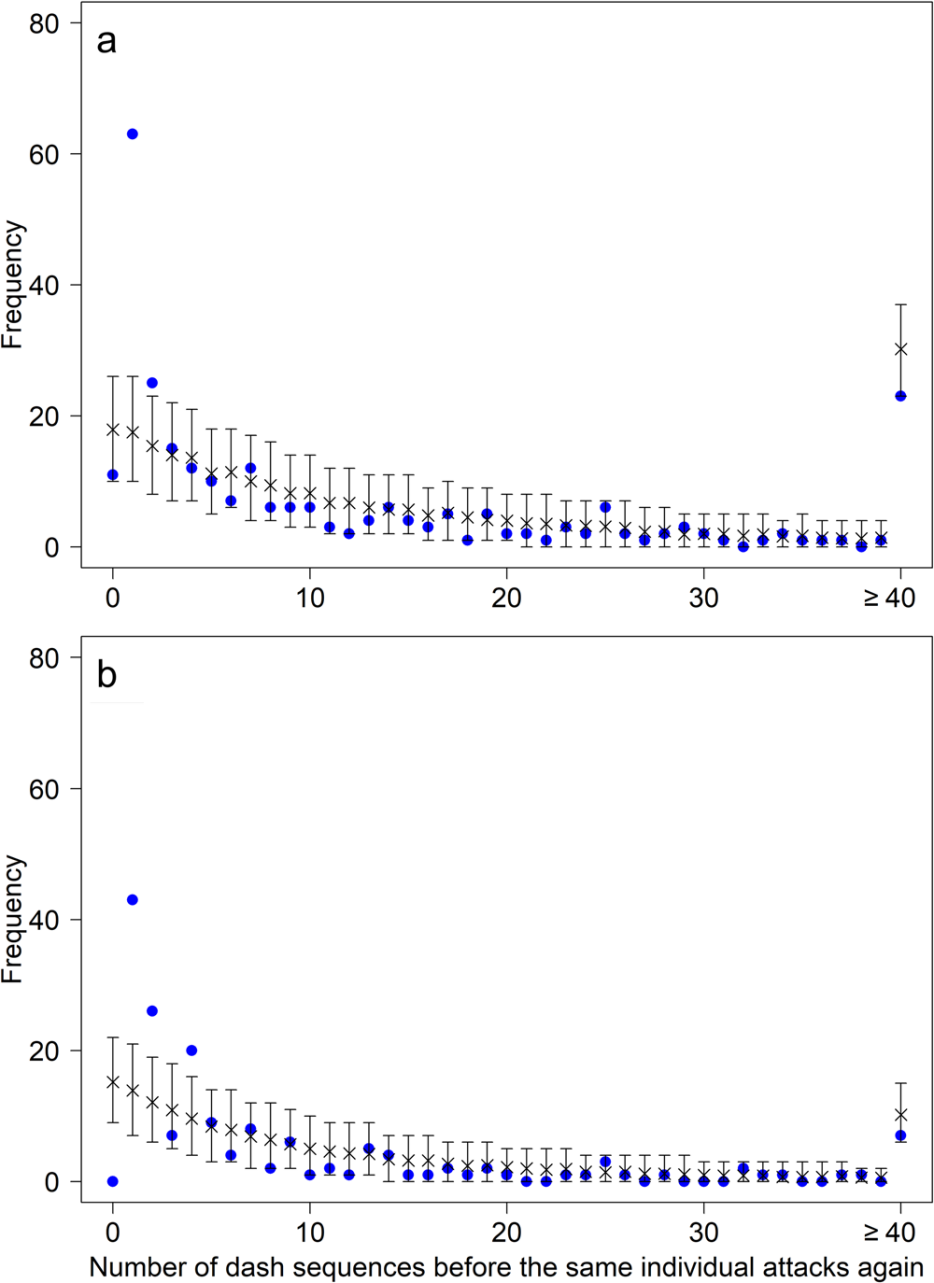
Numbers of dash sequences between two attacks of the same marlin. Observed () and expected (x) numbers of dash sequences of other marlin between two dash sequences of the same marlin in 2018 (**a**) and 2019 (**b**). Additionally, 2.5th and 97.5th percentiles for each point are shown (simulating 10^4^ repetitions). The outliers at x-axis value of 1 in both **a** and **b** show the occurrences of ABA dash sequences, i.e., where an individual marlin A, returns to attack immediately after any other marlin B’s single dash sequence. The final data point lies higher as it shows the data for the value 40 and above.

To explore this further, we investigated whether the probability of a given marlin (*A*) attacking immediately again after a single interruption by any other marlin (*B*) (*ABA* pattern), was higher than expected based on chance. This was indeed the case (Fig. 4a, b), further supporting the idea of competition. If marlin (*A*) stopped its dash sequence due to satiation or exhaustion, it would be unlikely to start a new dash sequence again immediately after a single interruption by a different marlin (*B*). In particular, the time spent handling and ingesting a sardine may have provided an opportunity for competitors to interrupt another marlin’s dash sequence (Supplementary Video 2). Despite this evidence for marlin interrupting each other, we did not observe any overt aggressive interactions such as chases or attempted slashes at each other. We did notice, however, that marlin which were trying to get back to the fish school after a tight turn may be forced to abandon their attack attempt because another marlin that was closer was already ahead of them.

No inter-individual variation was found in capture efficiencies (total captures divided by total dashes) over the entire hunt (2018: Fisher’s exact test, *p* = 0.19; 2019: *p* = 0.35).

### Marlin sizes

**Fig. 5:**
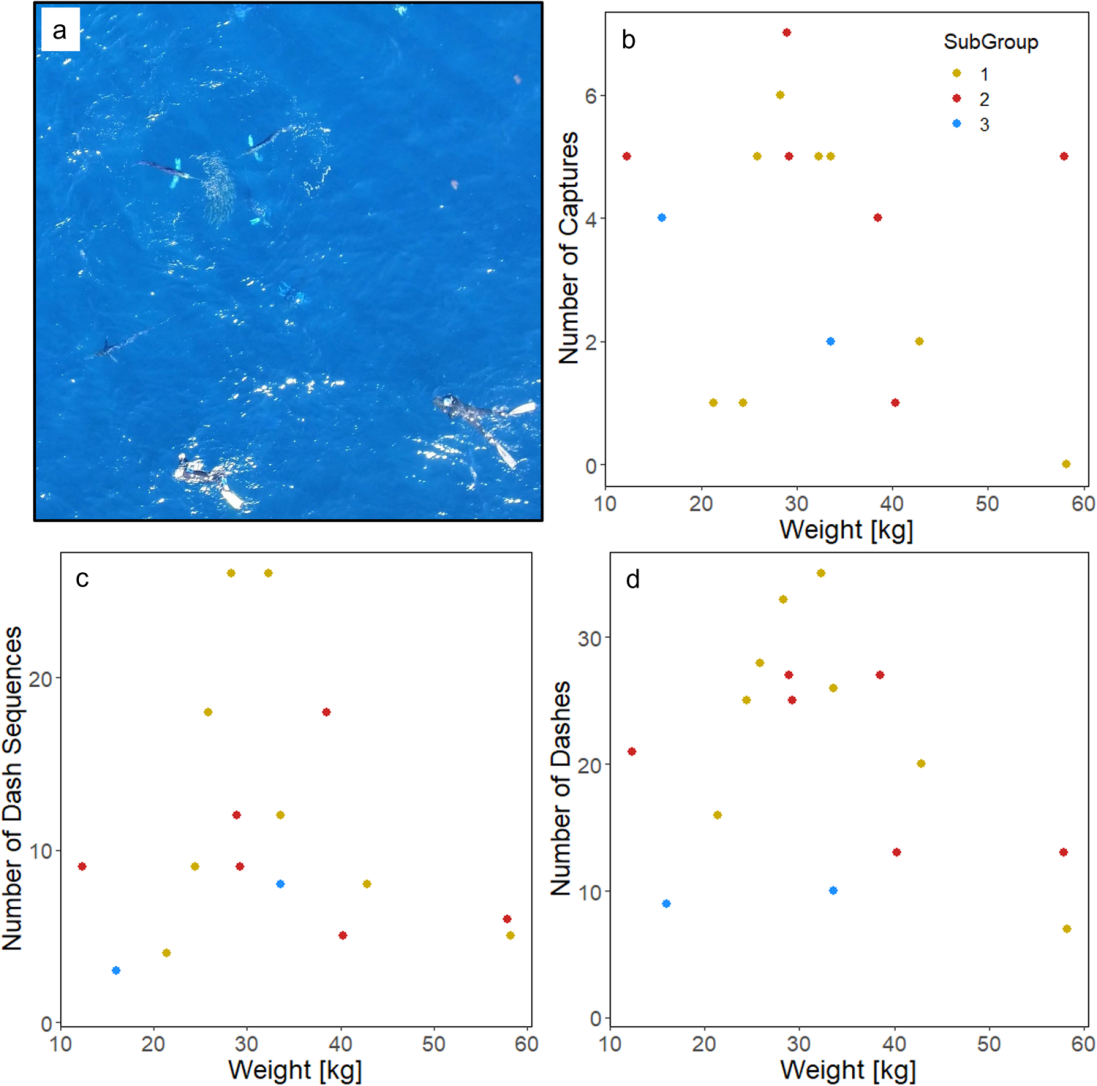
Number of captures, dash sequences and dashes in relation to marlin size. **a** Still frame from drone video in 2019 (image created by Felicie Dhellemmes). Still frames were used to size 16/20 individually identified marlin. There was no significant relationship between individual marlin weight (kg) and number of captures (**b**), number of dash sequences (**c**) or total number of dashes (**d**) performed by individual marlin. Marlin are colour-coded with respect to subgroup - 1/3 (), 2/3 () and 3/3 ().

Finally, in 2019 we used an unmanned aerial vehicle (Phantom 3 Pro, DJI) to record the marlin attacking the prey school^37^ (Fig. 5a). By matching up characteristic and easily identifiable events visible in both the underwater and drone video, we were able to identify individual marlin in the drone footage and take length measurements (eye-fork length) which we converted into estimated weights (kg) (*N* = 16, mean ± SD = 42.3 ± 14.2 kg, range 19.3–67.7 kg) (see Methods).

We found that there was no relationship between marlin weight and number of captures (Spearman correlation: *r* = −0.24, *p* = 0.38), number of dash sequences (Spearman correlation: *r* = 0.09, *p* = 0.75), or total number of dashes (Spearman correlation: *r* = −0.23, *p* = 0.38). There was also no discernible pattern of marlin weight and sub-grouping (Fig. 5b, c, d).

## Discussion

We report a previously unquantified form of prey division in group-hunting predators where there is frequent turn-taking for prey access. Through individual identification of striped marlin, we were able to show that the division of available prey was uneven and broadly in line with observations from some terrestrial predators with social hierarchies^2^. Although we cannot rule out dominance relationships as an explanation for the uneven prey division, the fission-fusion nature of striped marlin predator groups, the absence of correlation between marlin size and number of captures and the lack of aggression at the resource makes it seem unlikely that prey division related to dominance hierarchies within subgroups. We suggest that differences in prey division were largely a result of competition between differently motivated individuals, for which we have indirect evidence.

We observed that newly arrived marlin achieved more frequent access to the prey school. These new arrivals may on average be hungrier and more motivated than marlin which had already been feeding, suggesting that nutritional state could be a key determinant of prey division in this group-hunting system. However, the skew in prey captures shown in Fig. 3a, c was found at the level of sub-groups. Therefore, increased prey access by newly arrived marlin sub-groups cannot be the sole explanation for unequal prey division. Between-individual variation in nutritional state within sub-groups could contribute to relative levels of motivation and prey access, and therefore explain the patterns of unequal prey division. However, it is unknown how full each marlin’s stomach was before they arrived at the prey school. Sub-groups that arrived at the end of the hunt achieved more frequent access to the prey school (as per the other earlier arriving sub-groups) but caught less prey overall. This is because in total they performed fewer dashes and because of the absolute numbers of marlin present at the prey school and the available hunting time. For group-hunting teleost predators, many of which form non-kin related fission-fusion systems, prey division between predators often relates to differences in nutritional states. Front positions within the school provide greater intake, and these positions are preferentially taken up by hungry individuals^38–42^. However, the majority of these examples are from relatively small species that feed on plankton (they do not access the prey resource one at a time) and where grouping likely evolved primarily for anti-predator defence.

Our calculations of individual predator weight showed a large range (19.3–67.7 kg) which should conceivably relate to differences in nutritional requirements. Larger animals require greater intakes to sustain their metabolism, therefore we predicted that these animals would consume a disproportionate amount of the shared prey resource. However, marlin weight did not explain how the prey resource was divided between the predators, nor did it explain the patterns of sub-grouping. Our observations in the field suggest that marlin (which are visual hunters) may hunt schools of fish for as long as the available light allows, thus a relationship between marlin size and intake may play out over longer time periods. Beyond size and daily energetic requirements, however, the behaviour of individuals within groups of animals is affected by differences in hunger level related to the current nutritional state. Thus, acute differences in nutritional state between marlin, and therefore motivation levels (regardless of their overall size), may determine the attack dynamics in this system.

Indirect evidence for the role of motivational differences in prey division comes from the attack order results. The high frequency of *ABA* patterns suggests that marlin often interrupt each other’s attack sequences, but also shows that the original marlin *A* often managed to regain access immediately afterwards, suggesting that they were more motivated to continue the attack as compared to a random group member. A potential behavioural mechanism that could explain how highly motivated individuals gain more access than others could be that these marlins, compared to less motivated conspecifics, stay closer to the dynamically moving prey school manoeuvring themselves into the best positions to launch a new dash sequence. Drone footage provides anecdotal evidence for this explanation with some marlin coasting along at greater distance, whereas those near the school of prey perform many twists and turns. Future research tracking the movements of marlin in relation to each other, their prey intake, and the prey school is currently underway.

Some group-hunting species in the open ocean are known to typically surround their prey but attack one at a time^18^. This attack strategy could be related to the fact that large marine predators often swipe body parts to stun and kill prey (e.g. orca;^17^ billfish^20,36,43^), making it dangerous for conspecifics to be too close. This raises the question of whether there are differences in hunting skills and efficiency between predators that take turns, and whether this relates to the evolution of group-hunting. Kurvers et al. 2017^44^ found that more lateralised sailfish achieved greater capture efficiency and suggested that the presence of differently lateralised individuals in the same group would make it possible for sailfish to benefit from lateralisation while avoiding the cost of attack predictability. In marine mammals which often hunt within social groups with closed membership and repeated social interactions^45^, role differentiation in connection with group-hunting has been documented^17,46–49^. It has, however, been a considerable challenge to relate differences in roles to capture efficiencies^19^. We did not find differences in capture efficiency between individual marlin. However, we did measure statistically significant differences in the frequency of prey access.

Despite evidence for competition, a major characteristic that was evident in our study was the lack of aggression between marlin over an ephemeral resource. The school of prey was very difficult (often impossible) for individual predators to monopolise for any meaningful length of time. This condition potentially explains the reduction or complete loss of resource defence and aggressive interactions, which are also commonly reported from multi-species predator aggregations in the open ocean^33,50^. Future work could explore how open ocean inter-species predator interactions relate to prey division, and compare these to those found in terrestrial systems which are frequently characterised by high levels of competition and aggression.

In conclusion, by tracking the attack dynamics and capture successes of individually identified group-hunting marlin, we quantified how a shared prey resource was divided between the predators. Marlin displayed a version of turn-taking, where marlin would challenge each other for access to the prey school, whereupon a single marlin would make repeated dashes and temporally monopolise the resource before being interrupted by a conspecific. The resource was not shared equally between the predators and multiple different sources of evidence provide indirect support for motivationally-driven differences in prey access.

## Methods

### Study system and definitions of attack behaviour

We filmed group-hunting striped marlin (*Kajikia audax*) 10–30 km offshore Baja California, Mexico (N 24° 54.52-48.5’, W 112° 34.46-23.51’) in November 2018 and November 2019 as they attacked sardines (*Sardinops sagax caerula*, 2018: N = approx. *140; Scomber japonicus*, 2019: N = approx. 175; standard length = approx. 20 cm). We recorded (GoPro HERO4, 120 fps) marlin attacking the prey school continuously for 46 min in 2018 and 17 min in 2019 while snorkelling at 2–10 m distance. We identified individual marlin by characteristic markings such as lateral stripes and dorsal fin shape (Fig. 1a). For each individual, we scored when it attacked and whether or not the attack was successful (i.e. prey capture) (Fig. 1b). Marlin attacked schooling prey by ‘dashing’ through the school, often followed by a quick turn and reapproach of the school. Individuals may therefore make consecutive dashes at the prey school before a different marlin starts its own attack.

In 2019 we were able to partially record the hunt (7.5 / 17 min) with an unmanned aerial vehicle (Phantom 3 Pro, DJI) from a height of 30 m (2840 x 2160 px, 25 fps). By identifying the same characteristic events in the underwater and drone videos we were able to sync the two videos in time and therefore match the individual identities from the underwater video to the drone video. This allowed size measurements for 16/20 individual marlin. To size marlin, we took 6 eye-fork length (EFL) measurements from still frames of the video when the individual marlin was at the surface (i.e., when the dorsal or caudal fin was breaking the surface) and when its body was straight. Each EFL measurement (pixels) was calibrated by the known length of an observer’s diving fins that were present in each image to give a length in cm (*N* = 16, mean ± SD = 155.9 ± 15.5 cm, range 125.3–180.9 cm) which was converted to weight (kg) (*N* = 16, mean ± SD = 42.3 ± 14.2 kg, range 19.3–67.7 kg) using the equation $0.00000133263* {{EFL}}^{3.41344}$ ^51^. Our length measurements fit comfortably within range of other measurements of striped marlin taken in the area^52,53^ (albeit are around 15 cm (10%) smaller on average). We could not size four (out of twenty) individuals due to a lack of images of sufficient quality. These individuals only made eight dash sequences between them (range 1–3). The mean standard deviation of the six separate length measurements (within each of the 16 individuals) was 4.4 cm (range 1.2–8.9 cm).

The prey school video in 2019 is shorter, primarily because the hunting event (and the recording of data) was ended by the arrival of a tourist boat. However, in 2018, tourists did not interrupt the hunt and we witnessed the prey school being completely consumed by the striped marlin. Here, we could periodically count the number of sardines from still video frames to assess the accuracy of our data collection. Capture rate was relatively consistent throughout the hunt (Mean ± SE captures per minute: 2.27 ± 0.26, range 0–7; linear regression F_1,46_ = 4.1, *p* = 0.05). However, after approximately 2000 s of data collection, with approximately 50 sardines remaining, the prey school started to break up, making it increasingly difficult to record all captures. From this point on, our recorded captures began to lose precision compared to spot counts of sardine numbers from still video frames. In the remaining 800 s, we estimated that 25 captures were unaccounted for, however, up until this point (i.e. the first 2000 s) our estimates of how many sardines were captured from the school aligned well to the spot counts from still frames. We estimated only 5 unaccounted captures out of a total of 81 in the first 2067 s of data collection.

Therefore, when appropriate, we conducted two analyses for the 2018 data: one including all data (2789 s), and one including data up until we started to lose precision (specifically after 2067 s; hereafter called ‘subsample’). At this point, 23 individually identified marlin had attacked (out of 34), performing 198 dash sequences (out of 297), 519 individual dashes (out of 711) and 81 captures (out of 110). For the 2019 data, we conducted one analysis on all the available data.

### Marlin subgrouping

Marlin were individually identified when they attacked the sardines. The order in which individual marlin appeared in our recordings was used to investigate whether marlin arrived in groups. To do this, for each pair (*m*_*i*_, *m*_*i+1*_) of subsequently appearing marlin, we counted the number of dash sequences *d*_*i*_ of any marlin present between the appearances of *m*_*i*_ and *m*_*i+1*_. A large value of *d*_*i*_ (i.e. no new marlin appearing for a long period) followed by multiple smaller ones (i.e. several new marlin appearing within a short time) is indicative of a group arrival. To account for the increase of the values of *d*_*i*_ with the number of marlin present, we divided the values by the number *i* of marlin that were already present. If all marlin have the same chance of being the next one to attack and *i* marlin are present, a newly arriving marlin will, on average, have to wait for *i* dash sequences of other marlin before it performs its first attack. Finally, we looked for local maxima of these relative numbers *d*_*i*_/*i* in the ordered sequence of appearing marlin (Fig. 1c, Fig. S1b). More precisely, we regarded marlin *m*_*i+1*_ as the first member of a new group, if *d*_*i*_/*i* was greater than or equal to a certain threshold *h* and if *d*_*i*+1_/(*i* + 1) was smaller than or equal to min(1, *d*_*i*_/*i*). This means the minimum subgroup size is 2. This heuristic approach does not lead to an unambiguous segmentation. Depending on the value of *h*, we obtained 2 (1.3 ≤ *h* ≤ 3.5), 3 (1.1 ≤ *h* ≤ 1.2), or 4 groups (*h* = 1.0) in 2018 and 2 (2.1 ≤ *h* ≤ 3.2), 3 (1.6 ≤ *h* ≤ 2.0), or 4 groups (1.2 ≤ *h* ≤ 1.5) in 2019.

### Marlin access to resource - subgroups

To determine whether there were differences in prey access between marlin we compared the observed total number of dashes each marlin made to those expected based on the observed opportunities they had to attack. The expected numbers of dashes were computed under the assumption that each marlin had the same chance of performing the next dash, but only if they were present at the prey school. Since there was a strong indication that marlin arrived at different time periods during the hunt (Fig. 1b, c, Fig. S1a, b), marlin were deemed present after a member of their arriving group was observed making an attack. The number of opportunities in this computation equalled the number of observed dashes. Therefore, the sum of the expected values of all individuals also equalled the number of observed dashes. In our investigation we compared the observed and expected numbers of dashes for each newly arrived subgroup in the period from its arrival till the arrival of the next subgroup. In addition to the expected values, we computed 2.5th and 97.5th percentiles using a simulation with 10^6^ repetitions (Fig. 2a–c). This analysis assumed that all marlin stayed at the prey school until the end of the hunt. To investigate this assumption and check our results for robustness against some marlin leaving early, we analysed the lengths of time the marlin stayed at the prey school without performing an attack (Supplementary Material, Fig. S2, Table S2).

### Marlin access to resource - individual mean length of dash sequences

To investigate individual differences in mean length of dash sequences (mean number of consecutive dashes by a single marlin) we conducted a randomisation test (10^4^ steps), where in each step we permuted the observed lengths of dash sequences, while keeping constant the individual numbers of dash sequences. We accounted for the change in sequence lengths over time and only permuted the lengths within quarters of the hunt in 2018 (Fig. S3a) and in thirds of the hunt in 2019 (Fig. S3c). As a test statistic we used the variance of the mean dash sequence lengths of those individuals that performed at least 5 dash sequences (*N* = 21), which had a significantly large value (2018 - all data: variance =  1.01, *p* = 0.03; subsample: variance = 1.26, *p* = 0.03; 2019 - variance = 0.42, *p* = 0.02).

### Distribution of the lengths of dash sequences

The length of dash sequences followed a geometric distribution (Fig. S3a–d), characterized by a single parameter, the probability *p* of ending a dash sequence. This probability increased over time (Fig. S3a, Chi-squared test for trend in proportions, χ^2^ = 27.7, df = 1, *p* < 0.001) in the data of 2018. Therefore, we split the complete hunt into quarters (Fig. S3a, b), each of which contained 74 or 75 dashes. In the data of 2019, we did not find an increase, but for comparison with the data of 2018 we split the complete hunt into thirds (Fig. S3c, d).

### Analysis of *ABA* patterns

We explored the order in which individual marlin attacked for patterns that may be indicative of a mechanism determining when and why individual marlin attack. In the succession of identities of attacking marlin we found a significantly high number of *ABA* patterns compared to random expectations, where some marlin *A* immediately returned to attack after the dash sequence of any other marlin *B*. We analysed this using a randomisation test (10^4^ steps), where in each step we permuted the attacking order of marlin within each group. The results were significant: 2018 - observed number = 63, mean in the randomisations = 17.5 for 2 groups; 18.1 for 3 groups, and 22.6 for 4 groups, *p* < 0.001; subsample: observed number = 49, mean in the randomisations = 13.0 for 2 and 3 groups; 17.5 for 4 groups, *p* < 0.001. 2019 - observed number = 49, mean in the randomisations = 13.9 for 2 groups; 17.1 for 3 groups, and 17.9 for 4 groups, *p* < 0.001. We also analysed this pattern in a more general form by counting the number of dash sequences of other marlin that occurred between two dash sequences of the same marlin and compared this with randomised attacking orders (using the above-described randomisation scheme). The results are shown in Fig. 4a, b.

### Individual capture efficiencies

Our final analysis was to determine if there were individual differences in capture efficiencies. We conducted a Fisher’s exact test for count data on individual marlin capture efficiencies (using the raw data of each individual’s total number of captures and total number of dashes). We used Monte Carlo simulation with 10,000 replicates to simulate p-values.

### Statistics and reproducibility

Most statistical analyses (Chi-squared test, Spearman’s correlation test, Pearson’s correlation test, Fisher’s exact test) were performed in the R software environment version 3.5.0^54^ Further computations and randomisation tests are described in detail (including randomisation scheme and test statistic) in the respective subsections of the Methods.

The data presented is from observations of wild hunts, therefore it is impossible to reproduce exactly, however, we present data collected over 2 different years using the same methodology and show that the general patterns in the results are reproducible – the first year (2018) included 34 individually identified striped marlin that completed 711 attacks and 110 prey captures, and the second year (2019) included 20 individually identified striped marlin that completed 350 attacks and 58 prey captures. The same methodology can be used each year in October or November at the study location, in Baja California, Mexico, where the same behaviours can be observed (provided the population of striped marlin and prey fish are present).

### Reporting summary

Further information on research design is available in the Nature Research Reporting Summary linked to this article.

## Supplementary information

Supplementary Information

Description of Additional Supplementary Files

Supplementary Video 1

Supplementary Video 2

Reporting Summary

## Data Availability

Data^55^ is available on Dryad or by direct request to the author. Hansen, Matthew (2022), Mechanisms of prey division in a marine group-hunting predator, Dryad, Dataset, 10.5061/dryad.b2rbnzshx, https://datadryad.org/stash/share/yxsS4Lz0X-VbqTnI9qESmLZtACaGQjwU8sgYq2IFyCw
