# Supplementary material for: Mechanisms of prey division in striped marlin, a marine group hunting predator
Source: Commun Biol. 2022 Oct 31;5:1161. doi: 10.1038/s42003-022-03951-3 (PMC9622829; doi:10.1038/s42003-022-03951-3)
Supplement: Supplementary file 1 — Supplementary Information [file 42003_2022_3951_MOESM1_ESM.docx]

Supplementary Material

Duration of presence of marlin at the prey school

Our analysis of observed and expected numbers of dashes of individuals in newly arrived groups was based on the assumptions that all marlin stayed at the prey school until the end of the hunt. To investigate this assumption and check our results for robustness against some marlin leaving early, we performed an additional analysis. We first identified marlin that may have left the prey school before the end of the hunt based on the observed waiting times of all marlins. We then recomputed the results of Fig. 2 under the assumption that these marlin left the prey school immediately after their last dash (Fig. S2).

To quantify how long marlin were willing to wait at the prey school, we determined for each marlin *M* the maximum number of dashes *d_in between_* of other marlin that were observed between two dashes of *M*. Also, we determined the number of dashes *d_after_* of other marlin that were observed after the last dash of *M*. Then we compared the sets of values of *d_in between_* and *d_after_* (Table S2). In 2019 no value of *d_after_* was larger than the maximum value of *d_in between_*. Also, mean, median and standard deviation of the values of *d_in between_* were larger than those of *d_after_*, meaning that on average marlin were willing to wait between two dashes longer than the time between their last dashes and the end of our observations. In 2018, two values of *d_after_* (587 and 462 of individuals *K* and *F*, respectively) exceeded the values of *d_in between_* (max = 459). After setting these two values to 0, i.e. assuming *K* and *F* left immediately after their last dash, both sets had similar distributions (Table S2). Since the individual values of *d_in between_* and *d_after_* strongly depend on the individual arrival times, we also performed this analysis separately for individuals arriving during the first and second halves of the hunt. Again, the distributions were very similar (Table S2). Therefore, we concluded that most marlin were likely to stay till the end of the hunt. The assumption that the individuals *K* and *F* left early only slightly influenced the results regarding the observed and expected numbers of dashes of newly arriving marlin (Fig. S2). The trends were the same.

Table S1. Summary statistics of dashes and captures in total sample. Numbers in brackets show subsample (i.e. up until 2,067 seconds). In 2018 there were 34 individually identified marlin in the full sample and 23 in the subsample, in 2019 there were 20.

2018

|  | Number of Dash Sequences | Number of Individual Dashes | Dash Sequence Length | Number of Captures |
| --- | --- | --- | --- | --- |
| Total | 297 (198) | 711(519) | NA | 110 (81) |
| Mean | 8.7 (8.6) | 20.9 (22.6) | 2.4 (2.5) | 3.2 (3.5) |
| SE | 1.2 (1.3) | 2.8 (3.2) | 0.1 (0.2) | 0.6 (0.7) |
| Min | 1 (2) | 1 (3) | 1 (1) | 0 (0) |
| Max | 27 (23) | 64 (55) | 14 (14) | 11 (11) |

2019

|  | Number of Dash Sequences | Number of Individual Dashes | Dash Sequence Length | Number of Captures |
| --- | --- | --- | --- | --- |
| Total | 186 | 350 | NA | 58 |
| Mean | 9.3 | 17.5 | 1.9 | 2.9 |
| SE | 1.6 | 2.5 | 0.1 | 0.5 |
| Min | 1 | 1 | 1 | 0 |
| Max | 26 | 35 | 6 | 7 |

Table S2. Maximum, mean, median and standard deviation of the numbers of dashes of other marlin that were observed after the last dash of each marlin (*d_after_*) and the maximum numbers of dashes of other marlin that were observed between two dashes of the same marlin (*d_in between_*) for the complete hunt and its halves. *N* specifies the number of marlin arriving in the respective period. The row labelled with *d*_after_* contains the values from our robustness analysis regarding the time marlin were willing to stay at the prey school without performing an attack, where we assumed that two individuals (*K* and *F*) left immediately after their last dash.

|  | Complete hunt (*N* = 34) | | | | First half (*N* = 20) | | | | Second half (*N* = 14) | | | |
| --- | --- | --- | --- | --- | --- | --- | --- | --- | --- | --- | --- | --- |
|  | max | mean | median | sd | max | mean | median | sd | max | mean | median | sd |
| *d_after_* | 587 | 138.1 | 99.5 | 147.0 | 587 | 207.7 | 157.0 | 153.4 | 137 | 38.7 | 12.0 | 48.5 |
| *d*_after_* | 399 | 107.3 | 78.0 | 111.8 | 399 | 155.3 | 129.5 | 119.2 | 137 | 38.7 | 12.0 | 48.5 |
| *d_in between_* | 459 | 112.7 | 80.5 | 108.0 | 459 | 166.0 | 151.0 | 109.7 | 118 | 36.5 | 26.5 | 37.4 |

2018

2019

|  | Complete hunt (*N* = 20) | | | | First half (*N* = 15) | | | | Second half (*N* = 5) | | | |
| --- | --- | --- | --- | --- | --- | --- | --- | --- | --- | --- | --- | --- |
|  | max | mean | median | sd | max | mean | median | sd | max | mean | median | sd |
| *d_after_* | 269 | 61.8 | 43.5 | 63.0 | 269 | 71.1 | 50.0 | 68.7 | 76 | 33.8 | 34.0 | 32.2 |
| *d_in between_* | 297 | 85.3 | 59.0 | 85.4 | 297 | 108.1 | 63.0 | 86.4 | 63 | 16.6 | 7.0 | 26.1 |


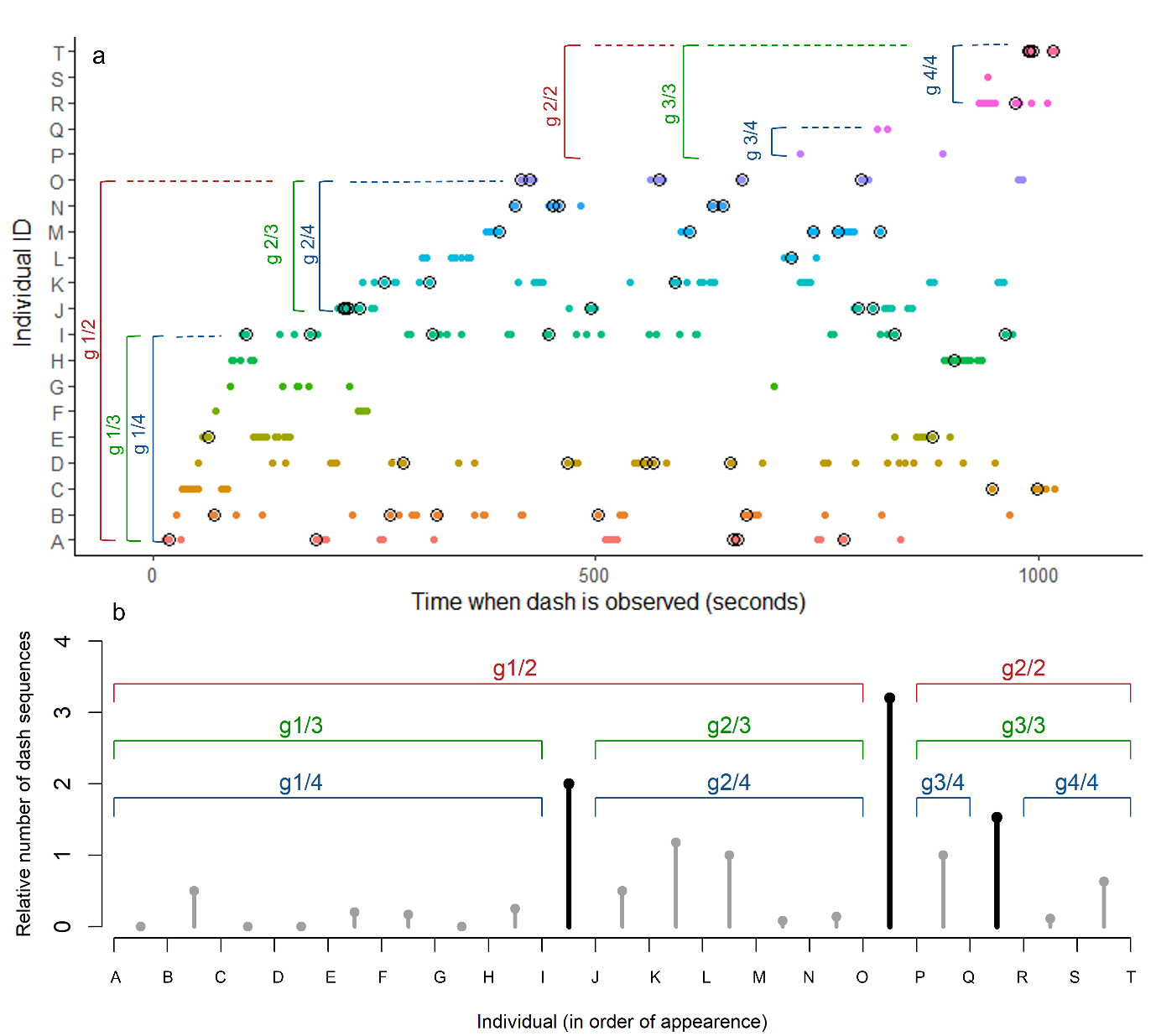


Fig. S1 (a) Each dot shows the timing of a dash by an individual marlin in 2019. Marlin are colour coded to differentiate between individuals. Dashes that resulted in a prey capture are encircled (○). Groups arriving at different time points are bracketed in different colours, with three potential scenarios: marlin arriving in two groups (red), in three groups (green), or four groups (blue). (b)The three potential sets of arriving groups of marlin in 2019 that can be derived from the numbers of dash sequences between the appearances of individual marlin, two groups (red), three groups (green) or four groups (blue).


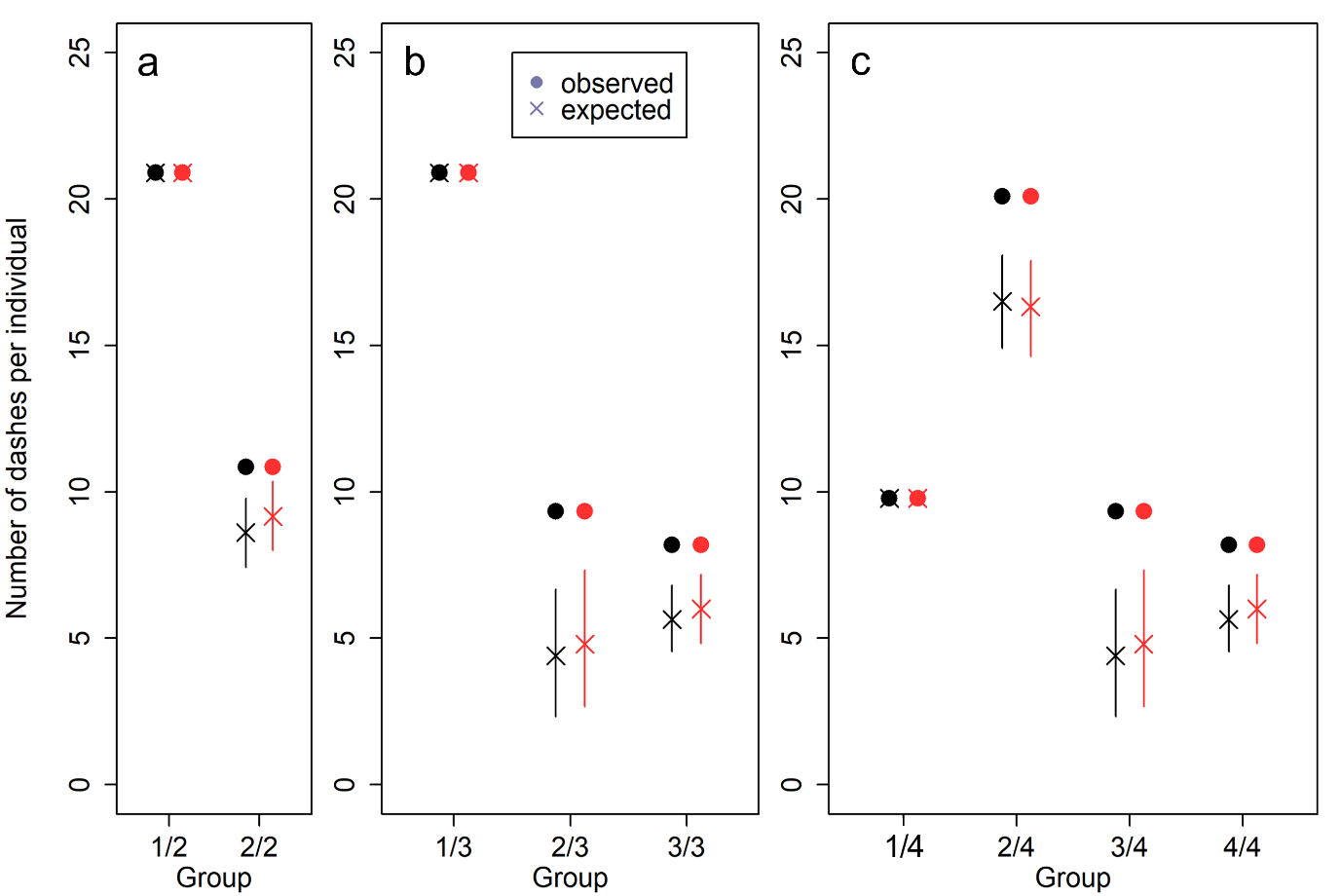


Fig. S2 (a-c) Observed (•) and expected (x) numbers of dashes per individual plus 2.5^th^ and 97.5th percentile for newly arrived groups in the period from their arrival until the arrival of the next subgroup for the data of 2018, assuming the marlin arrived at the prey resource sequentially in (a) two groups, (b) three groups or (c) four groups. Results based on the assumption that all marlin stayed at the prey school until the end of the hunt are shown in black and results of our robustness analysis (i.e. assuming that marlin *K* and *F* left after their last dash) in red.


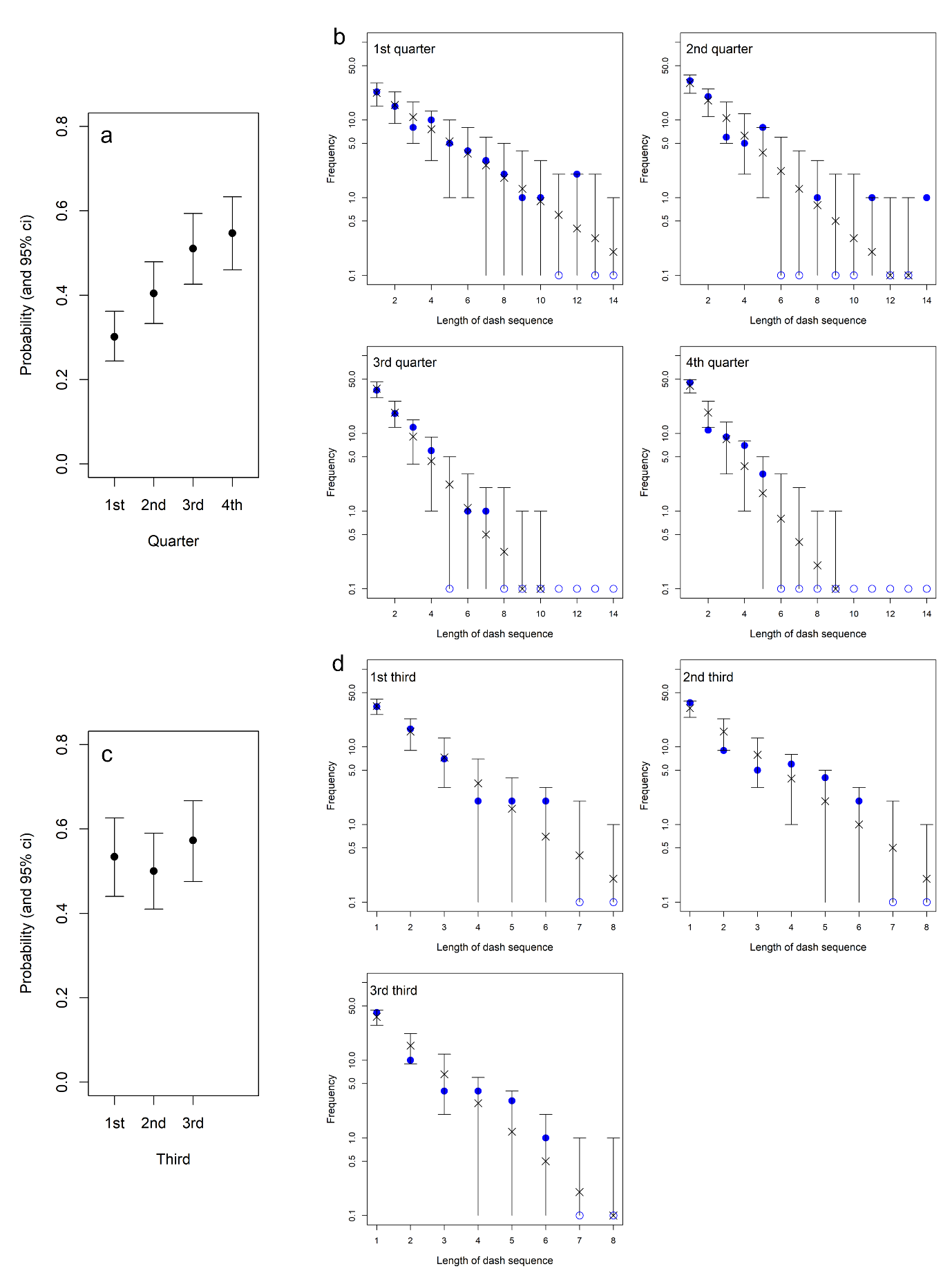


Fig. S3 (a, c) Estimated probabilities (plus 95% confidence intervals) of ending a dash sequence for the 4 quarters of the hunt (a - 2019) and 3 thirds of the hunt (c - 2019). (b, d) The observed lengths of dash sequences (•) fit approximately a geometric distribution (x) in each of the four quarters of the hunt (b - 2018) and 3 thirds of the hunt (d - 2019). Zero values, which cannot be displayed in a logarithmic plot, are indicated by hollow circles (o). The probability *p* of the geometric distribution was estimated from the observed values. Additionally, 2.5% and 97.5% percentiles for each point in this distribution are shown (simulating 10^4^ repetitions).
