# Supplementary material for: Mechanisms of prey division in striped marlin, a marine group hunting predator
Source: Commun Biol. 2022 Oct 31;5:1161. doi: 10.1038/s42003-022-03951-3 (PMC9622829; doi:10.1038/s42003-022-03951-3)
Supplement: Supplementary file 2 — Description of Additional Supplementary Files [file 42003_2022_3951_MOESM2_ESM.pdf]

Description of Additional Supplementary Files

File name: Supplementary Video 1
Description: Striped marlin dash sequence.

File name: Supplementary Video 2
Description: Striped marlin dash sequence with prey captures.
