# Supplementary material for: Mechanisms of prey division in striped marlin, a marine group hunting predator
Source: Commun Biol. 2022 Oct 31;5:1161. doi: 10.1038/s42003-022-03951-3 (PMC9622829; doi:10.1038/s42003-022-03951-3)
Supplement: Supplementary file 5 — Reporting Summary [file 42003_2022_3951_MOESM5_ESM.pdf]

nature portfolio

Corresponding author(s): Matthew James Hansen

Last updated by author(s): Aug 16, 2022

Reporting Summary

Nature Portfolio wishes to improve the reproducibility of the work that we publish. This form provides structure for consistency and transparency in reporting. For further information on Nature Portfolio policies, see our Editorial Policies and the Editorial Policy Checklist.

Statistics

For all statistical analyses, confirm that the following items are present in the figure legend, table legend, main text, or Methods section.

n/a	Confirmed	
☐	☒	The exact sample size (n) for each experimental group/condition, given as a discrete number and unit of measurement
☐	☒	A statement on whether measurements were taken from distinct samples or whether the same sample was measured repeatedly
☐	☒	The statistical test(s) used AND whether they are one- or two-sided<br>*Only common tests should be described solely by name; describe more complex techniques in the Methods section.*
☐	☒	A description of all covariates tested
☐	☒	A description of any assumptions or corrections, such as tests of normality and adjustment for multiple comparisons
☐	☒	A full description of the statistical parameters including central tendency (e.g. means) or other basic estimates (e.g. regression coefficient) AND variation (e.g. standard deviation) or associated estimates of uncertainty (e.g. confidence intervals)
☐	☒	For null hypothesis testing, the test statistic (e.g. F, t, r) with confidence intervals, effect sizes, degrees of freedom and P value noted<br>*Give P values as exact values whenever suitable.*
☒	☐	For Bayesian analysis, information on the choice of priors and Markov chain Monte Carlo settings
☐	☒	For hierarchical and complex designs, identification of the appropriate level for tests and full reporting of outcomes
☐	☒	Estimates of effect sizes (e.g. Cohen's d, Pearson's r), indicating how they were calculated

Our web collection on statistics for biologists contains articles on many of the points above.

Software and code

Policy information about availability of computer code

Data collection	VirtualDub v1.10.4 (stable) https://www.virtualdub.org/
Data analysis	R Core Team (2021). R: A language and environment for statistical computing. R Foundation for Statistical Computing, Vienna, Austria. URL https://www.R-project.org/.

For manuscripts utilizing custom algorithms or software that are central to the research but not yet described in published literature, software must be made available to editors and reviewers. We strongly encourage code deposition in a community repository (e.g. GitHub). See the Nature Portfolio guidelines for submitting code & software for further information.

Data

Policy information about availability of data

All manuscripts must include a data availability statement. This statement should provide the following information, where applicable:

- Accession codes, unique identifiers, or web links for publicly available datasets
- A description of any restrictions on data availability
- For clinical datasets or third party data, please ensure that the statement adheres to our policy

Data is available on Dryad or by direct request to the author.
Hansen, Matthew (2022), Mechanisms of prey division in a marine group-hunting predator, Dryad, Dataset, https://doi.org/10.5061/dryad.b2rbnzshx
https://datadryad.org/stash/share/yxsS4Lz0X-VbqTnI9qESmLZtACaGQjwU8sgYq2IFyCw

Field-specific reporting

Please select the one below that is the best fit for your research. If you are not sure, read the appropriate sections before making your selection.

☐ Life sciences ☐ Behavioural & social sciences ☒ Ecological, evolutionary & environmental sciences

For a reference copy of the document with all sections, see nature.com/documents/nr-reporting-summary-flat.pdf

Ecological, evolutionary & environmental sciences study design

All studies must disclose on these points even when the disclosure is negative.

Study description	Quantitative analysis of striped marlin hunting behaviour. Captures of prey fish (110 in 2018 and 58 in 2019) and attacks on prey fish (711 in 2018 and 350 in 2019) by individual marlin (34 in 2018, 20 in 2019) were recorded in 2018 and in 2019. The study shows how groups of striped marlin divide a sardine school between themselves.
Research sample	Groups of individually striped marlin (Kajikia audax) in 2018 and 2019. 34 striped marlin were individually identified in 2018 and 20 individuals in 2019. These individually striped marlin were potentially in different sub-groups (1-4) which is part of the manuscripts investigation and built into the manuscripts statistical analysis. The marlin captured 110 prey fish in 2018 and 58 in 2019. Striped marlin were chosen as the sample as they could be observed hunting and capturing individual sardines at the oceans surface. There were no manipulations, the data collected were observations of wild hunts. The sample represents the striped marlin population that aggregates off the coast of Baja California during October/ November.
Sampling strategy	Groups of marlin whose members could be individually identified from video were included in the analysis. Collecting data on wild hunts in the open ocean is very rare and this is the first study to collect data on capture and attack rates of individually identified members within a group. Thus, while the sample size may seem low it is of the highest detail yet recorded for a study of this type. The patterns in the data are very similar across the two years, and observations and calculations of more data from this system (that was unfortunately not of sufficient quality for individual identification of the striped marlin) also shows the same patterns of behaviour. This strongly suggests that the behaviours recorded in the sample represent the behaviours of the striped marlin population being studied..
Data collection	MJH, JK and FD searched for schools of sardines being predated upon by striped marlin by boat and by first locating diving birds. MJH then swam to the schools and recorded the hunting behaviour with underwater cameras. In 2019 FD also filmed the hunting behaviour with unmanned aerial vehicles.
Timing and spatial scale	The first two weeks of November in 2018 and 2019. We searched for sardine schools from sunrise to sunset as this was when marlin actively hunt.
Data exclusions	Data was not considered for analysis if marlin could not be individually identified from the video.
Reproducibility	The data presented is from observations of wild hunts, therefore it is impossible to reproduce exactly, however, we present data collected over 2 different years using the same methodology and show that the general patterns in the results are reproducible. The same methodology can be used each year in Oct/Nov at Baja California where the same behaviours can be observed (provided the population of striped marlin and prey fish are present).
Randomization	All marlin identified attacking the same prey school were considered part of the same hunting group. However, the sub-structure of this hunting group is part of the investigation of the manuscript.
Blinding	Data acquisition was blind in that the person collecting the capture and attack behavioural data did not know the identity of the marlin (as this was collected afterwards).

Did the study involve field work? ☒ Yes ☐ No

Field work, collection and transport

Field conditions	Open ocean 10-30km offshore
Location	10-30 km offshore Baja California, Mexico (N 24° 54.52-48.5’, W 112° 34.46-23.51’)
Access & import/export	All research was conducted in line with the laws and legislation of Secretaría de Medio Ambiente y Recursos Naturales (SEMARNAT), Mexico and complied to the guidelines regarding the treatment of animals in behavioural research
Disturbance	Data was not considered for analysis if it was disturbed by human interference (e.g. tourists or photographers not part of the study)

Reporting for specific materials, systems and methods

We require information from authors about some types of materials, experimental systems and methods used in many studies. Here, indicate whether each material, system or method listed is relevant to your study. If you are not sure if a list item applies to your research, read the appropriate section before selecting a response.

Materials & experimental systems

n/a	Involved in the study	
☒	☐	Antibodies
☒	☐	Eukaryotic cell lines
☒	☐	Palaeontology and archaeology
☐	☒	Animals and other organisms
☒	☐	Human research participants
☒	☐	Clinical data
☒	☐	Dual use research of concern

Methods

n/a	Involved in the study	
☒	☐	ChIP-seq
☒	☐	Flow cytometry
☒	☐	MRI-based neuroimaging

Animals and other organisms

Policy information about studies involving animals; ARRIVE guidelines recommended for reporting animal research

Laboratory animals	NA
Wild animals	Kajikia audax, Scomber japonicus and Sardinops sagax caerula were all observed in the wild. No manipulations or captures occurred. Animals were simply video recorded from a distance of 5-10m and left undisturbed so that they displayed natural behaviour.
Field-collected samples	The study did not contain samples collected from the field.
Ethics oversight	All research was conducted in line with the laws and legislation of Secretaría de Medio Ambiente y Recursos Naturales (SEMARNAT), Mexico and complied to the guidelines regarding the treatment of animals in behavioural research

Note that full information on the approval of the study protocol must also be provided in the manuscript.
